# Effect of childhood overweight on distal metaphyseal radius fractures treated by closed reduction

**DOI:** 10.1186/s13018-021-02336-2

**Published:** 2021-03-10

**Authors:** Yu Liu, Chunjie Liu, Dongmei Guo, Ning Wang, Ying Zhao, Dan Li

**Affiliations:** 1 Department of Pediatrics, Tangshan Workers Hospital, 27 Wenhua Road, Lubei District, Tangshan City, Hebei Province People’s Republic of China; 2 Department of Orthopedics, Tangshan Workers Hospital, Tangshan City, Hebei Province People’s Republic of China; 3 Department of Clinical Laboratory, Tangshan Workers Hospital, Tangshan City, Hebei Province People’s Republic of China

**Keywords:** Overweight, Obesity, Children, Redisplacement, Distal radius fractures

## Abstract

**Background:**

The medical community has recognized overweight as an epidemic negatively affecting a large proportion of the pediatric population, but few studies have been performed to investigate the relationship between overweight and failure of conservative treatment for distal radius fractures (DRFs). This study was performed to investigate the effect of overweight on the outcome of conservative treatment for DRFs in children.

**Methods:**

We performed a retrospective study of children with closed displaced distal metaphyseal radius fractures in our hospital from January 2015 to May 2020. Closed reduction was initially performed; if closed reduction failed, surgical treatment was performed. Patients were followed up regularly after treatment, and redisplacement was diagnosed on the basis of imaging findings. Potential risk factors for redisplacement were collected and analyzed.

**Results:**

In total, 142 children were included in this study. The final reduction procedure failed in 21 patients, all of whom finally underwent surgical treatment. The incidences of failed final reduction and fair reduction were significantly higher in the overweight/obesity group than in the normal-weight group (*P* = 0.046 and *P* = 0.041, respectively). During follow-up, 32 (26.4%) patients developed redisplacement after closed reduction and cast immobilization. The three risk factors associated with the incidence of redisplacement were overweight/obesity [odds ratio (OR), 2.149; 95% confidence interval (CI), 1.320–3.498], an associated ulnar fracture (OR, 2.127; 95% CI, 1.169–3.870), and a three-point index of ≥ 0.40 (OR, 3.272; 95% CI, 1.975–5.421).

**Conclusions:**

Overweight increases the risk of reduction failure and decreases the reduction effect. Overweight children were two times more likely to develop redisplacement than normal-weight children in the present study. Thus, overweight children may benefit from stricter clinical follow-up and perhaps a lower threshold for surgical intervention.

## Background

Distal radius fractures (DRFs) are the most common fractures in children. According to previous studies, DRFs account for 20 to 35% of all pediatric fractures [1–3]. These fractures are located in the epiphysis in 20% of children and in the metaphysis in 80% [4, 5]. Management of DRFs is controversial. Although the use of percutaneous pin fixation for completely displaced fractures is advocated by some scholars because of its good fixation effect, closed reduction with cast immobilization is still the most common treatment for these fractures [6–8]. However, the high incidence of redisplacement is a clear limitation of conservative treatment. Some authors have reported that the incidence ranges from 10 to 91% according to different definitions of redisplacement [9, 10]. In general, about one-third of patients may develop redisplacement during follow-up.

Childhood overweight or obesity continues to be a serious problem worldwide despite recent increases in awareness and prevention initiatives. According to recent studies, about one-third of children and adolescents in the USA are classified as either overweight or obese [11, 12]. This has resulted in increased awareness of the effects of overweight on the care of the pediatric population. Studies have shown that overweight in children is correlated with a higher incidence of extremity fractures and more complications following the treatment of some fractures [13, 14]. For conservatively treated fractures, successful reduction and maintenance of proper alignment can be difficult in overweight or obese children because of the larger soft tissue envelope. In this way, overweight may lead to reduction failure or loss of fracture reduction, increasing the possibility of further surgical treatment.

Few studies to date have been performed to investigate the relationship between overweight and failure of conservative treatment for DRFs, especially in children. Therefore, the present study was performed to analyze the effect of overweight on the outcome of conservative treatment. We hypothesized that overweight increases the risk of reduction failure and the incidence of fracture redisplacement.

## Materials and methods

### Patient population

After receiving approval from our institutional review board, we performed a retrospective study of children with displaced DRFs in our hospital from January 2015 to May 2020. The inclusion criteria were an age of 2 through 16 years and confirmation of distal metaphyseal radius fractures by X-ray or computed tomography images. A metaphyseal fracture was defined as a fracture proximal to and within 4 cm of the growth plate of the distal radius [6]. Patients with open fractures, epiphyseal injuries, concomitant upper extremity fractures, inadequate follow-up, or fractures initially treated by K-wires/plate fixation were excluded from this study.

### Treatment procedure and follow-up

All patients initially underwent closed reduction in the emergency room. When initial manipulation failed, additional reduction was performed under brachial block or general anesthesia in the operating room. If repeated reduction failed, the treatment was defined as failed final reduction and surgical treatment was planned. All reduction procedures were performed by experienced surgeons, and successful reductions were fixed by short-arm casts. Anteroposterior (AP) and lateral radiographs were taken before and after treatment.

**Fig. 1:**
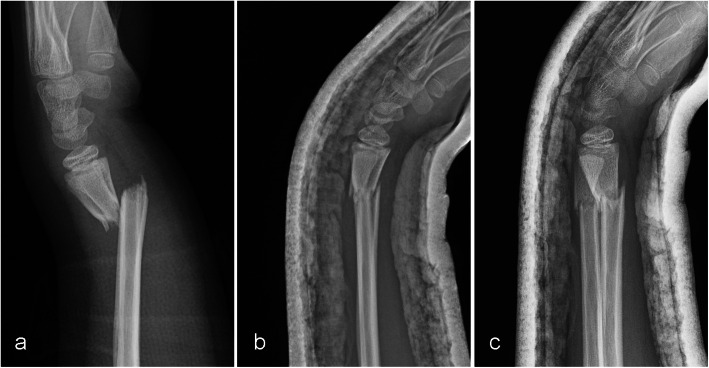
Lateral radiographs **a** before reduction, **b** after reduction, and **c** at the 1-week follow-up showing fracture redisplacement in an overweight child

The patients were followed up at 1, 2, 4, and 6 weeks after casting. Radiographs were taken at each routine follow-up visit. Redisplacement was defined as any modification from the initial AP or lateral radiograph after treatment (Fig. 1). After the fracture had healed, the cast was removed and wrist function exercises were started. The follow-up was finished when satisfactory wrist joint function was achieved.

### Parameter evaluation

Basic data including age, sex, height, and weight were collected from the electronic medical records. Each child’s body mass index (BMI) percentile was defined using sex-specific BMI-for-age charts established by the Centers for Disease Control and Prevention. Normal-weight children were defined as those with a BMI-for-age percentile (BMI percentile) of < 85, and overweight children were defined as those with a BMI percentile of 85 to < 95. Children with a BMI percentile of ≥ 95 were included in the obese cohort. These cutoff values were based on the Centers for Disease Control and Prevention definitions for children and teenagers [15–17].

The parameters obtained from radiographs included the distance to the epiphysis (distance from the radius fracture to the growth plate), whether an associated distal ulnar fracture was present, assessment of initial redisplacement, and the extent of reduction. The assessment of initial redisplacement included fracture translation and fracture angulation. The extent of reduction was classified as anatomic, good, or fair. Anatomic reduction was defined as complete anatomic fracture reduction with neither translation nor angulation, good reduction was defined as residual dorsal angulation of < 10° or residual translation of < 2 mm, and fair reduction was defined as angulation of 10 to 20° or translation of 2 to 5 mm [18].

The quality of immobilization was assessed using the three-point index as described by Alemdaroğlu et al. [18]. The corresponding measurements were obtained from radiographs after immobilization. This index was calculated as follows:

Three-point index = [(proximal radial gap + ulnar fracture site gap + distal radial gap)/contact between fracture fragments in AP plane] + [(proximal dorsal gap + volar fracture site gap + distal dorsal gap)/contact between fracture fragments in lateral plane]

Two independent observers assessed the radiological findings in a blinded manner, and the mean values were used for the data analysis.

### Data analysis

IBM SPSS Statistics for Windows, version 19.0 (IBM Corp., Armonk, NY, USA), was used to perform all statistical analyses. Categorical data were analyzed for significance by Fisher’s exact probability method, and numerical data were analyzed by the independent-samples *t* test. Variables that were demonstrated to be potentially associated with redisplacement after the univariate analysis (*P* < 0.10) were entered into the multiple logistic regression analysis, and a *P* value of < 0.05 was considered statistically significant.

## Results

After excluding patients who underwent initial wire or plate fixation, 142 children were included in this study. Among these patients, 101 (71.1%) were male and 41 (28.9%) were female. Their mean age at the time of fracture was 9.2 ± 3.1 years. According to the above-described criteria, 91 (64.1%) children were of normal weight, 32 (22.5%) were overweight, and 19 (13.4%) were obese (Table 1).

**Table 1:** Demographic data of children with displaced distal metaphyseal radius fractures

Variables	Values
Number of patients	142
Age (years)	9.2 ± 3.1
Gender	
Male	101 (71.1%)
Female	41 (28.9%)
Weight status	
Normal weight	91 (64.1%)
Overweight	32 (22.5%)
Obesity	19 (13.4%)
Conservative treatment outcome	
Successful reduction	121
Initial reduction	103 (85.1%)
Final reduction	18 (14.9%)
Failed reduction	21
Extent of reduction	
Anatomic	32 (26.4%)
Good	49 (40.5%)
Fair	40 (33.1%)
Number of patients with redisplacement	32
Time of redisplacement	
Within 1 week	23 (71.9%)
One week later	9 (28.1%)

**Fig. 2:**
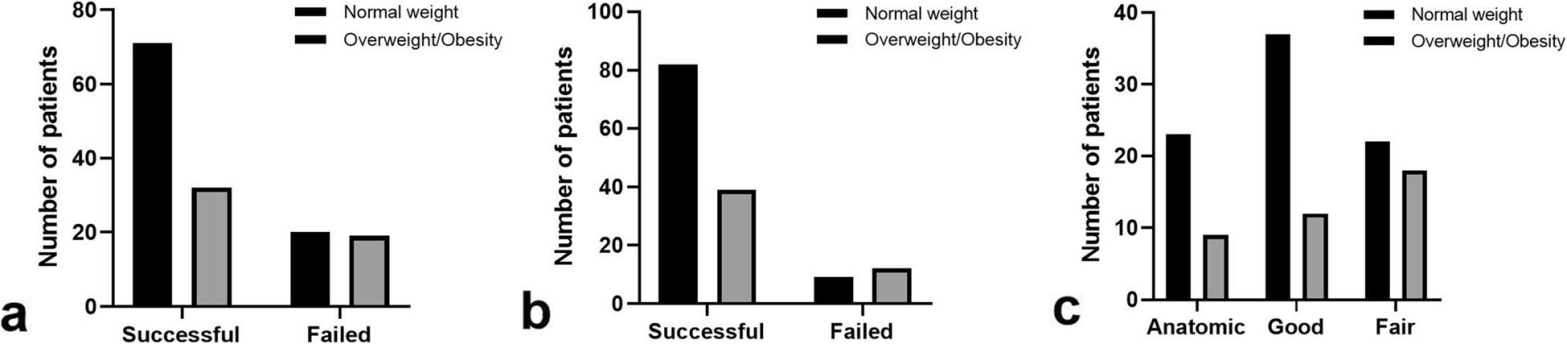
Numbers of normal-weight children and overweight/obese children with different **a** initial reduction results, **b** final reduction results, and **c** reduction extents

All patients initially underwent closed reduction under a local block in the emergency room. Initial reduction failed in 39 patients, all of whom required additional manipulations in the operating room. The final reduction procedure failed in 21 of these patients, all of whom then underwent surgical treatment. After conservative treatment, radiographs confirmed anatomical reduction in 32 (26.4%) patients, good reduction in 49 (40.5%), and fair reduction in 40 (33.1%). The patients’ data according to their different weight statuses in various treatment stages are shown in Fig. 2. The incidence of failed initial reduction was higher in the overweight/obesity group than in the normal-weight group (37.3% and 22.0%, respectively), but the difference was not statistically significant (*P* = 0.077). The incidences of failed final reduction and fair reduction were significantly higher in the overweight/obesity group than in the normal-weight group (*P* = 0.046 and *P* = 0.041, respectively).

During follow-up, 32 (26.4%) patients developed redisplacement after closed reduction and cast immobilization. Overall, 23 (71.9%) redisplacements occurred within 1 week after treatment, and 9 (28.1%) occurred 1 week later. No patients developed any serious complications such as compartment syndrome or permanent median nerve dysfunction.

We performed univariate and multivariate analyses to examine the effect of overweight on redisplacement. In the univariate analyses, we found that an overweight status (*P* = 0.002), the presence of an associated ulnar fracture (*P* = 0.004), initial translation of ≥ 50% (*P* < 0.013), and a high three-point index (*P* < 0.001) were potential risk factors associated with redisplacement after closed reduction, while other factors were not (*P* ≥ 0.10). The details of the univariate analyses are listed in Table 2. In the further multivariate logistic regression, the three factors associated with the incidence of redisplacement during follow-up were overweight/obesity [odds ratio (OR), 2.149; 95% confidence interval (CI), 1.320–3.498], an associated ulnar fracture (OR, 2.127; 95% CI, 1.169–3.870), and a three-point index of ≥ 0.40 (OR, 3.272; 95% CI, 1.975–5.421) (Table 3).

**Table 2:** Univariate analysis of data in children with and without redisplacement during follow-up

Potential risk factors	With redisplacement	Without redisplacement	*P* value
Number of patients	32	89	
Age (years)	8.8 ± 3.6	9.5 ± 3.3	0.317
Gender			
Male	24	64	0.820
Female	8	25	
Weight status			
Normal weight	14	68	0.002
Overweight/obesity	18	21	
Distance from epiphysis (mm)	19.2 ± 7.8	20.1 ± 6.6	0.530
Associated ulna fracture			
Yes	15	17	0.004
No	17	72	
Initial translation			
< 50%	17	69	0.013
≥ 50%	15	20	
Initial angulation			
< 20°	18	64	0.125
≥ 20°	14	25	
Anatomical reduction			
Yes	5	27	0.160
No	27	62	
Three-point index	0.57 ± 0.19	0.46 ± 0.13	< 0.001

**Table 3:** Multivariate analysis of risk factors associated with redisplacement in children with displaced distal metaphyseal radius fractures

	*P* value	Odds ratio	95% CI
Overweight/obesity	0.004	2.149	1.320–3.498
Associated ulna fracture	0.030	2.127	1.169–3.870
Initial translation ≥ 50%	0.108	1.669	0.924–3.013
Three-point index ≥ 0.40	< 0.001	3.272	1.975–5.421

*CI*, confidence interval

## Discussion

Closed reduction and casting is a widely accepted treatment for displaced distal metaphyseal radius fractures. Although the initial reduction failed in some patients in the present study, the overall rate of successful reduction was 85.8%. However, the rate of redisplacement was 26.4% during follow-up. The incidences of failed final reduction and fair reduction were significantly higher in the overweight/obesity group than in the normal-weight group. The univariate and multivariate analyses showed that overweight/obesity, an associated ulnar fracture, and a high three-point index were independent risk factors associated with the incidence of redisplacement. Overweight children were two times more likely to develop redisplacement than normal-weight children.

The medical community has recognized overweight and obesity as an epidemic negatively affecting a large proportion of the pediatric population across the nation, introducing new physiological and social problems. Although most of the associated health concerns involve endocrine abnormalities and an increased risk of cardiovascular disease later in life [19, 20], clinicians should also be aware of the orthopedic issues associated with childhood overweight, including an increased risk of fracture and greater fracture severity [21–23]. Growing numbers of reports are detailing higher rates of complications associated with surgical and conservative treatments in overweight children [24, 25]. Several studies have revealed a relationship between obesity and DRFs [26, 27], but few have focused on the pediatric population.

The large soft tissue envelope in the forearm of overweight children creates more difficulty in achieving effective reduction. In the present study, the incidence of failed initial reduction was higher in the overweight/obesity group than in the normal-weight group, but the difference was not statistically significant. We believe that this lack of significance may have been due to the relatively small sample size. The incidence of failed final reduction was significantly higher in the overweight/obesity group than in the normal-weight group, confirming our hypothesis that overweight increases the risk of reduction failure. In a previous study of pediatric both-bone forearm fractures, Okoroafor et al. [28] also found that a higher percentage of overweight and obese children than normal-weight children required surgical intervention after failure of nonsurgical management. Moreover, we found that the incidences of failed final reduction and fair reduction were significantly higher in the overweight/obesity group than in the normal-weight group, which supports the conclusion of Auer et al. [29] that obese children with distal radius and forearm fractures achieve poorer reduction.

Fracture redisplacement in the present study usually resulted from fracture instability or weak external fixation. Overweight/obesity, an associated ulnar fracture, and a high three-point index were three potential risk factors associated with the incidence of fracture redisplacement. An associated ulnar fracture increases the instability of fractures and thus increases the risk of redisplacement. A high-quality cast following reduction is important, and a high three-point index representing poorly molded cast can lead to unsatisfactory external fixation [30]. We speculate that the forearms of overweight or obese children have a disproportionate muscle-to-adipose tissue ratio, and the increased distance gives the cast less of a mechanical advantage to control the angulation or translation of the fracture. Children with these risk factors require a significantly more frequent follow-up visit.

This study has several limitations. First, this study was affected by the limitations inherent to its retrospective observational design. Second, this study only included patients with distal metaphyseal radius fractures. The results are not applicable to children with DRFs of the epiphysis. Third, a limited number of risk factors were investigated in the present study. Inclusion of other factors in future studies may provide more valuable information. Finally, the radiological measurements were performed without considering interobserver or intraobserver reliability, and the measurements could have been influenced by minor differences in the patients’ forearm positioning during the radiographic examinations. Similar studies with a prospective design, inclusion of more factors, and reliable measurements are still necessary.

## Conclusion

We found that the rate of successful reduction for displaced distal metaphyseal radius fractures was 85.8% and that the rate of redisplacement was 26.4% during follow-up. Overweight increases the risk of reduction failure and reduces the reduction effect. Overweight/obesity, an associated ulnar fracture, and a high three-point index were demonstrated to be independent risk factors associated with the incidence of redisplacement. Overweight children are two times more likely to develop redisplacement than normal-weight children. These patients may benefit from stricter clinical follow-up and perhaps a lower threshold for surgical intervention.

## Data Availability

Not applicable.

## Abbreviations

DRFs: Distal radius fractures
CDC: Centers for Disease Control
AP: Anteroposterior
BMI: Body mass index
